# Association between polymorphisms of thymidylate synthase gene 5′- and 3′-UTR and gastric cancer risk: meta-analysis

**DOI:** 10.1042/BSR20160273

**Published:** 2016-12-23

**Authors:** Ao Mo, Yongliang Zhao, Yan Shi, Feng Qian, Yingxue Hao, Jun Chen, Shiwei Yang, Yuxing Jiang, Ziyan Luo, Peiwu Yu

**Affiliations:** * Department of General Surgery and Center of Minimal Invasive Gastrointestinal Surgery, Southwest Hospital, Third Military Medical University, Chongqing 400038, China

**Keywords:** gastric cancer, meta-analysis, polymorphism, thymidylate synthase gene

## Abstract

Gastric cancer is the most common cancer and the most frequent cause of cancer death worldwide. Several studies have identified the role of thymidylate synthase (TS) 5′- and 3′-UTR and gastric cancer susceptibility; however, the results still remain inconclusive. The purpose of this meta-analysis was to reinvestigate this correlation. In the present study, online databases were searched to retrieve relevant articles published between January 2000 and 2016. The odds ratio (OR) and 95% confidence interval (CI) were employed to calculate the strength of association. Overall, a total of 13 articles were screened out, including 2382 gastric cancer patients and 3171 healthy controls. We found that polymorphisms of TS 5′-UTR 2R (double repeats)/3R (triple repeats) of a 28-bp sequence (11 articles) and 3′-UTR del6/ins6 (seven articles) were not significantly associated with increased risk of gastric cancer. Subgroup analysis by ethnicity showed that 2R allele and 2R/2R genotype in TS 5′-UTR were associated with gastric cancer susceptibility in Caucasian and African populations; del6 allele, del6/del6 and del6/ins6 genotypes were correlated with gastric cancer in Caucasian population. In conclusion, our result suggested that TS polymorphisms might be the risk factors for gastric cancer risk in Caucasian population, although this association needs further study, and future large-scale researches are still required.

## INTRODUCTION

Gastric cancer (or stomach cancer) is a disease in which malignant (cancer) cells develop from the lining of the stomach [1]. It remains a major health problem worldwide, and may spread from the stomach to other parts of the body, particularly the liver, lung, bone, lining of the abdomen and lymph nodes [2,3]. Gastric cancer is the fifth leading cause of cancer and the third leading cause of death worldwide making up 7% of cases and 9% of deaths [4]. According to the cancer statistics, there are approximately 26370 estimated new cases and 10730 estimated deaths in the United States in the year 2016 [5]. Infection with bacteria called *Helicobacter pylori* is a major cause of gastric cancer [6], and other risk factors include gender, cigarette smoking, atrophic gastritis and partial gastrectomy [7,8]. Up to now, gastric cancer remains a major diagnostic and therapeutic challenge [9,10]. The diagnosis is based on conventional white light endoscopy findings, and the prognosis depends on its stage [11]. Surgical resection is the only treatment modality that is potentially curative, but the majority of patients still relapse following resection [12]. In addition, treatment of advanced gastric cancer is controversial and there is no standard regimen for first- or second-line chemotherapy (CT) [13]. Detection of this disease in its early stage is helpful to improve the treatment outcome. Therefore, identifying some biomarkers associated with gastric cancer susceptibility can be used in predicting this disease and guiding the therapeutic strategies.

The development of gastric cancer is a complex and multistep process [14], and the mechanism under this disease is still relatively unknown. A small number of patients may have a genetic predisposition syndrome, and the total number of genome alterations was estimated at 4.18 for gastric cancer [15]. It is characterized by genomic instability that could be either microsatellite instability or chromosomal instability [16], involving multiple genetic and epigenetic alterations in oncogenes, tumour suppressor genes, DNA repair genes, cell-cycle regulators and signalling molecules [17,18]. Thymidylate synthase (TS) gene, located on chromosome 18p11.32, is the critical rate-limiting enzyme in the *de novo* synthesis of dTMP from deoxyuridine monophosphate (dUMP), which is required for DNA synthesis and repair [19]. It is a target for major chemotherapeutic drugs, and its expression is associated with tumour aggressiveness and poor prognosis [20]. In patients with advanced gastric cancer, TS has been identified as a prognostic marker, and high TS expression is associated with worse overall survival [21]. Several polymorphisms in the TS UTRs, which may influence TS mRNA transcription, message stability or protein expression, have been identified [22]. Two functionally important and ethnically diverse polymorphisms are the most extensively studied: TS enhancer region (TSER), a tandem-repeat polymorphism, which contains triple (3R) or double (2R) repeats of a 28-bp sequence in TS 5′-untranslated enhanced region, may be involved in modulation of TS mRNA expression [23]; and a 6-bp ins/del polymorphism on the 3′-UTR (position TS1494, del6 or ins6), which may influence mRNA stability [24]. The presence of the 3R compared with 2R 28-bp repeat sequence has been shown to enhance mRNA transcription and protein expression in *in vitro* and *in vivo* studies [25].

Although several molecular epidemiological studies were conducted to investigate the association between these two TS polymorphisms and gastric cancer risk. However, the results from different studies are divergent to some extent. Moreover, there is a marked geographical variation, with the highest rates reported in East Asia, South America and Eastern Europe and the lowest rates in the United States and Western Europe [26,27]. Therefore, we conducted this meta-analysis to clarify this issue and to obtain a more precise estimation of the relationship between TS polymorphisms and gastric cancer susceptibility.

## MATERIALS AND METHODS

### Study identification

We searched the electronic databases of Chinese language (CNKI and Wanfang) and English language (PubMed, Emabase and Medline) to retrieve relevant articles published between January 2000 and 2016. The MeSH terms: “gastric cancer or gastric carcinoma or stomach cancer”, “thymidylate synthase or TS gene” and “polymorphism or variant” as well as their combinations were used as the searching words. The corresponding Chinese version was used in the Chinese databases. References of related articles were manually searched. When the same authors or laboratories reported this issue on the same population, only the recent full-text articles were included.

### Inclusion criteria

The included studies must meet the following criteria: (1) case-control studies evaluating the effect of TS 5′-UTR and 3′-UTR polymorphisms in gastric cancer risk; (2) the diagnosis of gastric cancer should be made from gastroscopy or surgical biopsy reviewed by an experienced pathologist, and histology should be reported according to the World Health Organization criteria [28]; (3) controls should be unrelated, ethnically matched, healthy individuals; (4) the results were presented in odds ratio (OR) with its 95% corresponding confidence interval (CI); and (5) genotype information in patients and controls were available to extract.

### Data extraction

Two authors independently assessed the information of each included study. Any disagreement was resolved by discussion with a third expert. Each item should be able to reach a final consensus. The following information was extracted from each article: first author, published year, country, mean age, sample size, genotyping methods, genotype distribution and Hardy–Weinberg equilibrium (HWE) in controls.

### Statistical analysis

The association between TS polymorphisms and gastric cancer risk was measured by pooled OR with 95% CI. The significance of the pooled OR was determined by the *Z*-test, and a *P* value less than 0.05 was considered significant. The allelic model (M compared with m), homozygote model (MM compared with mm), heterozygote model (Mm compared with mm), dominant model (MM+Mm compared with mm) and recessive model (MM compared with Mm+mm) were calculated. The *I*^2^ test and the Q-statistic test were used to determine the between-study heterogeneity. The fixed-effect model was used when the effects were assumed to be homogeneous (a *P*-value more than 0.10 for the Q-test and *I*^2^ less than 50% for the *I*^2^ test), otherwise, the random-effect model was employed. Funnel plot asymmetry was used to assess the publication bias. Analyses were performed using the software Review Manager 5.3 (Oxford). All *P*-values were two-sided.

## RESULTS

### Characteristics of eligible studies

**Figure 1:**
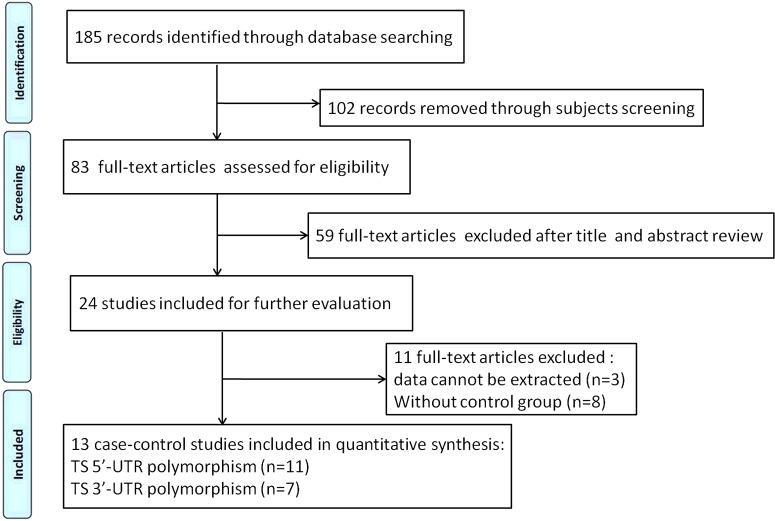
Flow chart of study selection process in this meta-analysis

Using the combined search, we first identified 185 relevant references. After applying the inclusion criteria, 13 studies were finally screened out into this meta-analysis, including 2382 gastric cancer patients and 3171 healthy controls. Figure 1 represents the searching process. The 13 studies (one was written in Chinese language [29] and twelve in English language [30–41]) were performed in six countries (China, Korea, Tunisia, Turkey, U.S.A. and Italy). Nine case-control studies were from Asian population, three studies were from Caucasian population, whereas only one study was from African population. The sample size ranged from 86 to 810. Polymorphisms of TS gene were measured by the PCR-restriction fragment length polymorphism (PCR-RFLP). The genotype distribution in controls was in accordance with HWE except studies conducted by Gao et al. [31], Baroudi et al. [39] and Sumen et al. [41]. The detailed characteristics of included studies were summarized in Table 1. The information of alleles and genotypes of TS 5′-UTR and 3′-UTR polymorphisms was presented in Table 2.

**Table 1:** Main characteristics of included studies in this meta-analysis –, not available

			Mean age		Sample size		Genotyping
First author	Year	Country	Cases	Controls	Cases	Controls	Method
Gao, C.M.	2004	China	36–81	36–81	155	223	PCR-RFLP
Graziano, F.	2004	Italy	59 (30–83)	58 (33–77)	134	139	PCR-RFLP
Zhang, J.H.	2004	China	55.0 (105)	51.3 (10.7)	233	348	PCR-RFLP
Tan, W.	2005	China	–	–	231	492	PCR-RFLP
Wang, L.D.	2005	China	58 (40–80)	56 (46–76)	129	315	PCR-RFLP
Zhang, Z.D.	2005	China	58.7 (9.7)	58.1 (10.6)	322	337	PCR-RFLP
Yang, L.	2008	China	–	–	60	170	PCR
Jung, H.	2010	Korea	59.3 (12.4)	45.8 (16.0)	300	100	PCR
Yim, D.J.	2010	Korea	58.26 (12.75)	57.18 (12.65)	318	280	PCR-RFLP
Baroudi, O.	2014	Tunisia	56 (30–70)	47.02 (20–80)	52	88	PCR-RFLP
Pan, X.	2014	China	22–76	18–55	31	200	PCR-RFLP
Sumen, I.C.	2014	Turkey	59 (37–79)	53 (25–75)	38	48	PCR-RFLP
Shen, R.	2015	U.S.A.	59.7 (12.7)	59.1 (11.2)	379	431	PCR-RFLP

**Table 2:** Alleles and genotypes of TS 5′- and 3′-UTR polymorphisms in this meta-analysis

First author 5′-UTR	Cases					Controls					
	3R/3R	3R/2R	2R/2R	3R	2R	3R/3R	3R/2R	2R/2R	3R	2R	HWE
Graziano, F.	38	76	18	152	112	31	74	31	136	136	0.303
Zhang, J.H.	148	76	8	372	92	223	107	13	553	133	0.97
Tan, W.	157	60	14	374	88	337	137	18	811	173	0.385
Wang, L.D.	81	39	8	201	55	201	108	6	510	120	0.139
Zhang, Z.D.	217	101	19	535	139	203	107	12	513	131	0.649
Yang, L.	31	26	3	88	32	103	54	8	260	70	0.789
Jung, H.	199	91	10	489	111	60	30	10	150	50	0.135
Yim, D.J.	211	89	18	511	125	194	79	7	467	93	0.755
Baroudi, O.	18	8	26	44	60	26	4	58	56	120	0.000
Pan, X.	20	8	3	48	14	146	48	6	340	60	0.405
Sumen, I.C.	7	18	13	32	44	9	6	33	24	72	0.000
**3′-UTR**	**ins6/ins6**	**ins6/del6**	**del6/del6**	**ins6**	**del6**	**ins6/ins6**	**ins6/del6**	**del6/del6**	**ins6**	**del6**	
Gao, C.M.	10	80	65	100	210	18	121	84	157	289	0.018
Graziano, F.	39	73	22	151	117	62	59	18	183	95	0.505
Zhang, J.H.	24	105	104	153	313	34	155	159	223	473	0.671
Zhang, Z.D.	53	143	141	249	425	30	139	153	199	445	0.846
Yim, D.J.	29	130	159	188	448	19	121	140	159	401	0.294
Pan, X.	3	10	18	16	46	22	90	88	134	266	0.888
Shen, R.	144	163	72	451	307	192	190	49	574	288	0.847

**Table 3:** Summary of pooled ORs with CI of TS 5′- and 3′-UTR polymorphisms in gastric cancer risk in this meta-analysis *N*, number of included studies; F, the fixed-effect model; R, the random-effect model.

				Test of association		Test of heterogeneity		
SNP	Group	Comparisons	*N*	OR (95% CI)	*P*	Ph	*I*^2^	Model
5′-UTR	Total	2R compared with 3R	11	0.97 (0.81, 1.14)	0.68	0.01	55%	R
		2R/2R compared with 3R/3R		1.08 (0.68, 1.71)	0.76	0.003	63%	R
		3R/2R compared with 3R/3R		1.01 (0.88, 1.17)	0.86	0.43	2%	F
		2R/2R+3R/2R compared with 3R/3R		1.01 (0.88, 1.15)	0.90	0.74	0%	F
		2R/2R compared with 3R/2R+3R/3R		0.98 (0.58, 1.63)	0.93	<0.0001	73%	R
	Asians	2R compared with 3R	8	1.08 (0.95, 1.21)	0.24	0.23	25%	F
		2R/2R compared with 3R/3R		1.42 (0.83, 2.42)	0.19	0.02	59%	R
		3R/2R compared with 3R/3R		0.99 (0.86, 1.15)	0.94	0.83	0%	F
		2R/2R+3R/2R compared with 3R/3R		1.04 (0.90, 1.20)	0.59	0.67	0%	F
		2R/2R compared with 3R/2R+3R/3R		1.41 (0.83, 2.40)	0.21	0.01	60%	R
	Non-Asians	2R compared with 3R	3	0.66 (0.51, 0.85)	0.001	0.044	0%	F
		2R/2R compared with 3R/3R		0.54 (0.34, 0.88)	0.01	0.84	0%	F
		3R/2R compared with 3R/3R		1.82 (0.62, 5.30)	0.27	0.05	67%	R
		2R/2R+3R/2R compared with 3R/3R		0.78 (0.52, 1.18)	0.24	0.87	0%	F
		2R/2R compared with 3R/2R+3R/3R		0.44 (0.29, 0.67)	0.0001	0.31	16%	F
3′-UTR		del6 compared with ins6	7	1.09 (0.90, 1.33)	0.37	0.003	70%	R
		del6/del6 compared with ins6/ins6		1.12 (0.72, 1.76)	0.61	0.002	70%	R
		Ins6/del6 compared with ins6/ins6		1.00 (0.73, 1.38)	0.98	0.05	53%	R
		del6/del6+del6/ins6 compared with ins6/ins6		1.05 (0.74, 1.49)	0.80	0.009	65%	R
		del6/del6 compared with del6/ins6+ins6/ins6		1.14 (0.90, 1.45)	0.27	0.03	56%	R
	Asians	del6 compared with ins6	5	0.94 (0.83, 1.06)	0.31	0.15	40%	F
		del6/del6 compared with ins6/ins6		0.78 (0.59, 1.04)	0.10	0.23	29%	F
		ins6/del6 compared with ins6/ins6		0.77 (0.57, 1.03)	0.08	0.58	0%	F
		del6/del6+del6/ins6 compared with ins6/ins6		0.77 (0.59, 1.02)	0.07	0.36	8%	F
		del6/del6 compared with del6/ins6+ins6/ins6		0.98 (0.83, 1.15)	0.79	0.29	19%	F
	Caucasians	del6 compared with ins6	2	1.39 (1.17, 1.66)	0.0002	0.64	0%	F
		del6/del6 compared with ins6/ins6		1.96 (1.35, 2.82)	0.0003	0.98	0%	F
		ins6/del6 compared with ins6/ins6		1.43 (0.85, 2.42)	0.18	0.08	67%	R
		del6/del6+del6/ins6 compared with ins6/ins6		1.44 (1.13, 1.85)	0.003	0.17	47%	F
		del6/del6 compared with del6/ins6+ins6/ins6		1.68 (1.20, 2.36)	0.003	0.41	0%	F

### Correlation of TS 5′-UTR 2R/3R polymorphism in gastric cancer susceptibility

**Figure 2:**
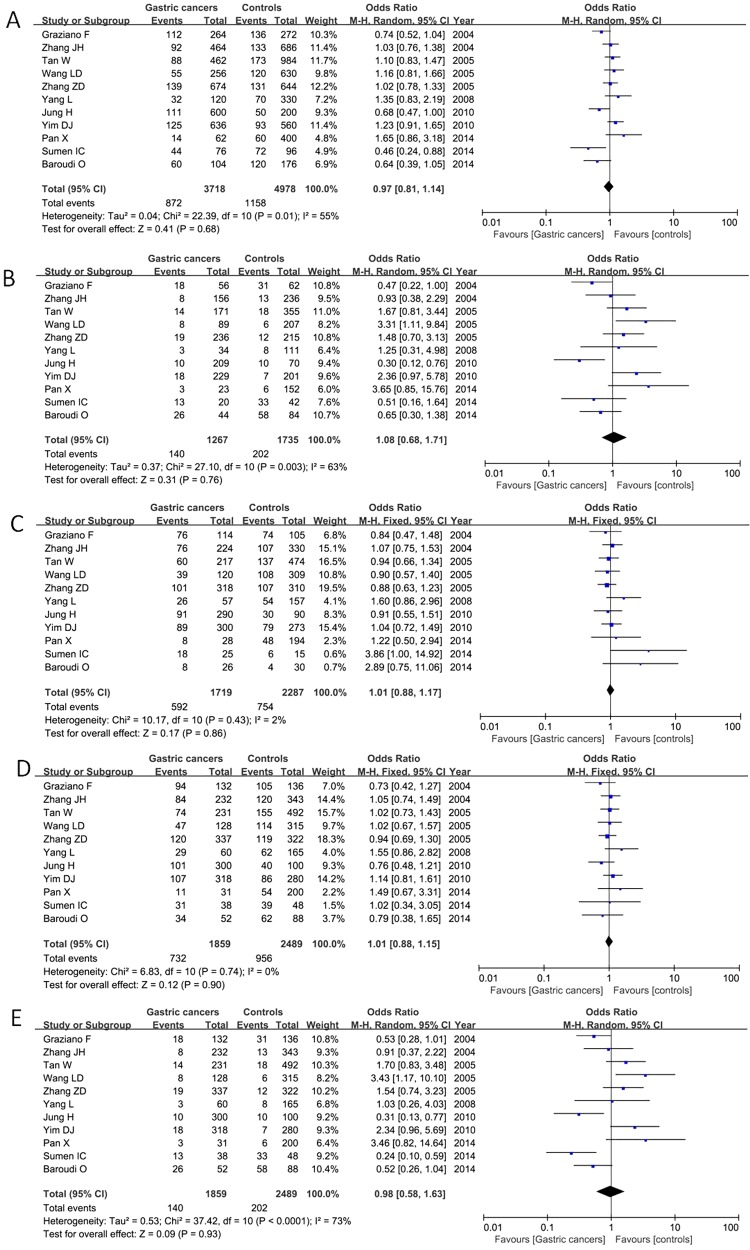
Meta-analysis of the correlation between TS 5′-UTR polymorphism and gastric cancer susceptibility under the allelic model (**A**), homozygote model (**B**), heterozygote model (**C**), dominant model (**D**) and recessive model (**E**)

The results of this meta-analysis were listed in Table 3. Eleven articles including 1859 patients and 2489 controls were eligible for pooling OR data. The between-study heterogeneity was calculated, the fixed-effect model was used in the heterozygote model and the dominant model, whereas the random-effect model was performed in other comparison models. Analyses of the 11 relevant studies showed that there was no obvious association between TS 5′-UTR 2R/3R polymorphism and gastric cancer risk under any genetic models as shown in Figure 2.

**Figure 3:**
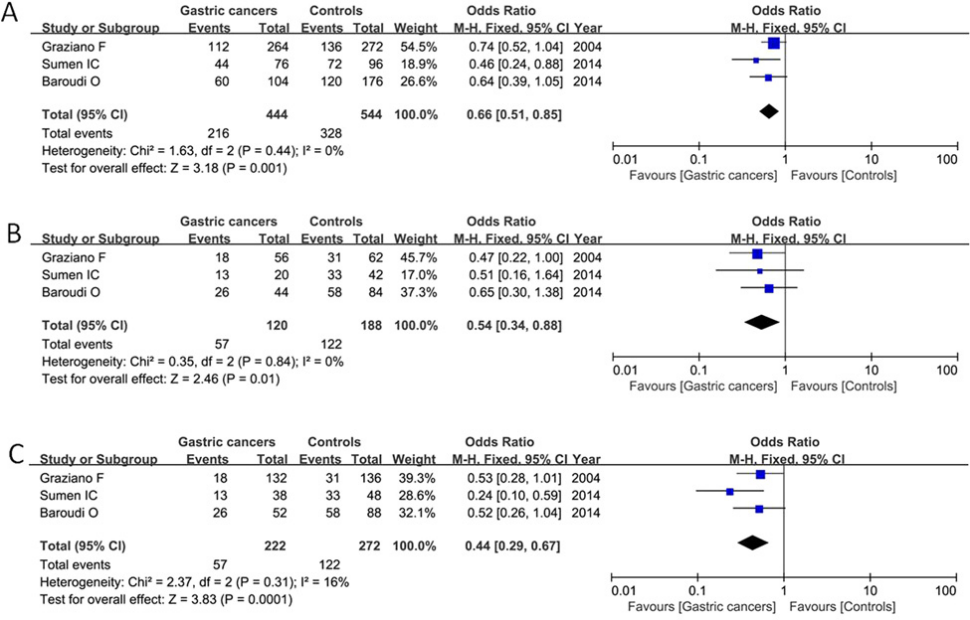
Forest plot of TS 5′-UTR variant and gastric cancer risk under the allelic model (**A**), homologous model (**B**), and recessive effect (**C**) in the fixed-effect model in non-Asian populations (Caucasian and African)

Subgroup analysis was conducted based on ethnicity including Asian population and non-Asian population (Caucasian and African). Our result detected that 2R of TS 5′-untranslated enhanced region contributed to gastric cancer risk in non-Asian population under the allelic model (2R compared with 3R: OR=0.66, 95% CI=0.51–0.85, *P*=0.001), homozygote model (2R/2R compared with 3R/3R: OR=0.54, 95% CI=0.34–0.88, *P*=0.01) and recessive model (2R/2R compared with 3R/2R+3R/3R: OR=0.44, 95% CI=0.29–0.67, *P*=0.0001) in the fixed-effect model as shown in Figure 3. For the Asian population, there was no obvious association between TS 5′-UTR 2R/3R polymorphism and gastric cancer susceptibility under the five comparison models (Table 3).

### Correlation of TS 3′-UTR del6/ins6 polymorphism in gastric cancer susceptibility

**Figure 4:**
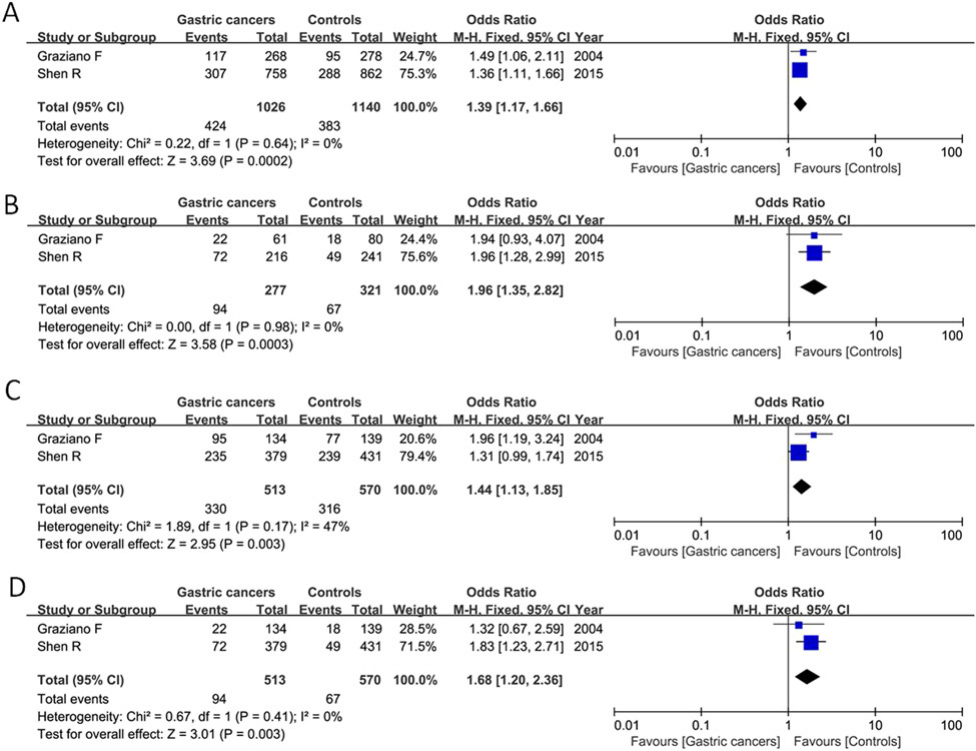
Forest plot of pooled OR with 95% CI for TS 3′-UTR polymorphism and gastric cancer risk under the allelic model (**A**), homozygote model (**B**), dominant model (**C**) and recessive model (**D**) in Caucasian population

Seven articles contained 1587 gastric cancer patients and 1943 controls. Significant heterogeneity among included studies was detected, and the random-effect model was employed. Our result found that the TS 3′-UTR del6/ins6 variant was not associated with gastric cancer risk under any genetic models (Table 3). Subgroup analysis by ethnicity showed that TS 3′-UTR del6/ins6 variant was significantly related with gastric cancer risk under the allelic model (del6 compared with ins6: OR=1.39, 95% CI=1.17–1.66, *P*=0.0002), homozygote model (del6/del6 compared with ins6/ins6: OR=1.96, 95% CI=1.35–2.82, *P*=0.0003), dominant model (del6/del6  + del6/ins6 compared with ins6/ins6: OR=1.44, 95% CI=1.13–1.85, *P*=0.003) and recessive model (del6/del6 compared with del6/ins6+ins6/ins6: OR=1.68, 95% CI=1.20–2.36, *P*=0.003) in Caucasian population as shown in Figure 4. No correlation was detected between this genetic variant and gastric cancer risk in Asian population.

### Sensitivity analysis and publication bias

**Figure 5:**
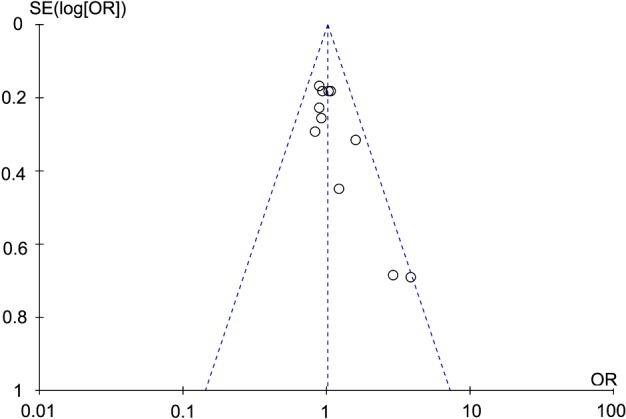
Funnel plot of TS 5′-UTR polymorphism in gastric cancer risk under the heterogeneous model

We conducted the sensitivity analysis to verify whether our results were affected by each included study. Our result indicated that single study could not influence the pooled OR qualitatively, suggesting that the result was stable. Funnel plot was used to assess publication bias, and the shape of the funnel plots was symmetrical, indicating no publication bias in this meta-analysis as shown in Figure 5.

## DISCUSSION

In this meta-analysis, we totally screened out 13 relevant articles concerning TS 5′-UTR 2R/3R and 3′-UTR del6/ins6 polymorphisms. Our result found that both these two variants were not associated with gastric cancer risk. Subgroup analysis by ethnicity showed that 2R of TS 5′-UTR 2R/3R and del6 of TS 3′-UTR del6/ins6 variants were associated with increased risk of gastric cancer risk in Caucasian population. Our result was not consistent with three previous meta-analyses: one was conducted by Yang et al. [42] that contained six included articles and suggested that 2R of TYMS 5′-UTR 2R/3R contributed to gastric cancer risk in the Asian population, one was conducted by Lu et al. [43] that suggested that the 3R variant of TS 5'-untranslated enhanced region 2R/3R polymorphism contributed to gastric cancer risk in the Caucasian population, and the last one was conducted by Zhuang et al. [44] that showed that polymorphisms in the 5′-UTR and 3′-UTR of the TS gene might be associated with gastric cancer susceptibility.

Gastric cancer is a prevalent yet heterogeneous disease. The role of genetic predisposition to gastric cancer has been suggested by epidemiological studies [45]. Individual genetic susceptibility may be critical in a variety of processes relevant to the tumorigenesis of gastric cancer, such as the cell apoptotic pathway, cell proliferation ability, the intrinsic variability of DNA repair processes, the inflammatory response and the functioning of carcinogen detoxification and antioxidant protection [46]. Identifying the relevant genes can be used as a tool to search for genetic variations of the disease's genes and susceptibility, thus to increase understanding of this disease's mechanism [47].

The folate metabolic pathway is involved in the synthesis and methylation of DNA, and low folate levels were shown to be associated with an increased risk of gastric cancer [48]. TS is one of the key enzymes that involved in the folate metabolism. Variants of TS gene differ not only biologically but also functionally in their ability to alter TYMS activation on folate metabolism [49]. Furthermore, TS holds promise as a prognostic biomarker because of its role as the molecular target of 5-FU, a commonly used chemotherapeutic agent in gastric cancer [50]. High TS gene expression in tumours was associated with enhanced benefit from post-operative adjuvant S-1 treatment in gastric cancer [51], and might predict drug resistance and adverse prognosis in patients with advanced stages treated with FU-based CT [52]. TS expression was also associated with CT response, progression-free survival and overall survival in advanced gastric cancer patients treated with capecitabine alone CT [53]. Moreover, TS mRNA level in plasma can mirror tumour TS mRNA level, and both of them can be used to predict raltitrexed sensitivity in gastric cancer [54].

Several studies have identified the role of TS polymorphisms in gastric cancer risk; however, the results still remain inconclusive. Shen et al. [40] found that the del6/del6 genotype of TS 3′-UTR was associated with significantly increased gastric cancer risk, Sumen et al. [41] suggested that 3R allele, 2R/3R and 3R/3R genotypes were risk factors for gastric cancer, whereas Araújo et al. [55] did not obtain a significant relationship between TS 5′-UTR 2R/3R and 3′-UTR del6/ins6 polymorphisms and gastric cancer susceptibility. Studies have also shown that TS polymorphisms might be associated with other cancers’ risks such as colon cancer [56], lung cancer [57] and oral squamous cell carcinoma [58]. In addition, genetic variation in TS gene may affect carcinogenesis through the regulation of gene expression, the status of the dNTP pool and drug sensitivity. Gao et al. [59] first reported that TS 3′-UTR ins6/ins6 genotype could predict the poor survival of advanced gastric cancer patients treated with capecitabine plus paclitaxel. Huang et al. [60] found that the polymorphisms of TS 3′-UTR del6/ins6 might be potential prognostic factor in gastric cancer patients treated with 5-FU-based adjuvant CT. Kim et al. [61] demonstrated that TS genotyping could be of help in predicting toxicity in oral fluoropyrimidine-based CT in advanced gastric cancer patients.

Several limitations were presented in this meta-analysis. Firstly, the number of included studies for subgroup analysis was small, which might affect the accuracy of our result. Secondly, the stages of patients with gastric cancer could not be extracted from the included articles due to the lack of sufficient data. Thirdly, gene–gene interaction should be addressed in the future meta-analysis for the study of combined polymorphisms, instead of single low-penetrance variations in susceptibility that may lead to a high-risk classification for a specific population [62]. Lastly, other factors, such as gender, smoking and drinking should be considered as well in the future researches.

In conclusions, our meta-analysis suggested that 2R of TS 5′-UTR 2R/3R and del6 of TS 3′-UTR del6/ins6 might contribute to gastric cancer risk in the Caucasian population. However, future large-scale studies with more ethnicities are still needed to further investigate this association.

## Abbreviations

CI: confidence interval
CT: chemotherapy
del6: 6-bp deletion
dNTP: deoxyribonucleoside triphosphates
5-FU: 5-fluorouracil
HWE: Hardy–Weinberg equilibrium
ins6: 6-bp insertion
MeSH: Medical Subject Headings
OR: odds ratio
PCR-RFLP: PCR-restriction fragment length polymorphism
Ph: *p*-value of heterogeneity
2R: double repeats
3R: triple repeats
S-1: TS-1, Taiho Pharmaceutical
SNP: Single nucleotide polymorphism
TS: thymidylate synthase
TYMS: thymidylate synthase
